# Evaluating the effectiveness of the Kidogo model in empowering women and strengthening their capacities to engage in paid labor opportunities through the provision of quality childcare: a study protocol for an exploratory study in Nakuru County, Kenya

**DOI:** 10.1057/s41599-022-01260-y

**Published:** 2022-07-15

**Authors:** Kenneth Okelo, Margaret Nampijja, Patrick Ilboudo, Ruth Muendo, Linda Oloo, Sylvia Muyingo, Elizabeth Mwaniki, Nelson Langat, Silas Onyango, Florence Sipalla, Patricia Kitsao-Wekulo

**Affiliations:** African Population and Health Research Center–Kenya, Nairobi, Kenya

**Keywords:** Development studies, Health humanities

## Abstract

Worldwide, there is a wide gap between what women can contribute to the economy and what they actually contribute. One of the main barriers to women’s engagement in the labor market and productivity at work is the societal expectation that they should take care of their children in addition to meeting the demands of employment. Furthermore, those in informal employment face difficulties due to long working hours and environments that are not appropriate for childcare. To address this, Kidogo runs an innovative “Hub & Spoke” model for low-income communities. Here, we present a study protocol aimed at evaluating whether the provision of quality childcare opportunities for working women through the Kidogo model is feasible and acceptable and whether it contributes to improvements in their incomes and productivity at work. The study reported in this protocol which is currently ongoing, employed a quasi-experimental design with two study arms: primary caregivers who use childcare services were recruited into the intervention (*n* = 170) and comparison groups (*n* = 170). Both groups are being followed up for one year. We are using a mixed-methods approach. Appropriate statistical methods including a difference-in-differences (DID) estimator will be used to analyze the effects of the intervention. We expect that the intervention will improve the quality of childcare services which in turn will improve the incomes of the center providers. We expect that providing improved childcare services will enhance women’s economic empowerment.

**Trial registration:** PACTR202107762759962.

## Introduction

The overall purpose of the proposed project is to evaluate the effectiveness of the Kidogo model which aims at empowering women economically through the provision of quality childcare services. This goal is based on the evidence that women spend disproportionately more time on unpaid work such as childcare and household chores than men (International Labor Organization, 2008). Studies from North America, Europe, and Latin America indicate that women’s childcare responsibilities significantly impede their participation in the paid labor force. Similar barriers have been observed in sub-Saharan Africa (Doughman et al., 2017). Interventions that directly provide alternative childcare options and give women greater freedom of choice and action enable them to meaningfully contribute to economic production processes. For instance, a recent study illustrated that provision of subsidized childcare services resulted in women being more likely to engage in economic activities than those who did not have access to such services (Doughman et al., 2017). The quality and cost of paid childcare services also influence the decision to take their children to childcare and engage in employment, or stay at home and look after their children (Clark et al., 2018).

The challenges faced by women in balancing childcare and paid work are compounded in low-income urban contexts where employment opportunities are limited, and fragmented social networks often mean that mothers cannot rely on kin to provide childcare support. Further, women in informal employment face challenges such as long working hours and environments that are not conducive to carrying their babies to work or breastfeeding (Kimani-Murage et al., 2015). In awareness of this gap and to have a sustainable solution, Kidogo, a social enterprise established in 2014 (www.kidogo.co), seeks to improve access to high-quality, affordable early childhood care and education to support the healthy growth and development of young children living in urban informal settlements.

Kidogo runs an innovative “Hub & Spoke” model for low-income communities through a two-fold approach. First, by establishing best practice early childhood development (ECD) “hubs” with a holistic curriculum, the program nurtures the healthy growth and development of children, with caregivers trained on the principles of ECD. Secondly, Kidogo works to improve the quality of local childcare centers, referred to as “spokes” through its micro-franchising program with “mamapreneurs.” Mamapreneurs are local women running informal childcare centers within the community who are given a ‘business-in-a-box,’ including relevant training materials, resources, and support. In this way, Kidogo builds the capacity of facility owners to enhance self-employment opportunities.

The Kidogo hubs operate as model centers of excellence for the spokes, and act as mini-research and development (R & D) labs to test innovations and demonstration sites for the community. The hubs serve 50–100 children aged between six months and three years for eight to ten hours during the working day. Kidogo provides the basic building blocks for young children to thrive, known as “The Kidogo Way,” which includes safe, stimulating environments, trained ECD caregivers, and its proprietary play-based curriculum and health, nutrition and protection program. On weekends and during holiday months, Kidogo hubs turn into training centers for the mamapreneurs. Since 2014, Kidogo has grown to support over 130 such childcare centers serving 2886 children daily in 12 different informal settlements across Nairobi.

Addressing the burden of childcare for mothers in informal settlements enhances their opportunities to participate in gainful employment, which, in turn, may contribute to improved nurturing care for their children. By improving the quality, consistency, and availability of childcare and pre-school services in communities, Kidogo is able to give young children the best start to life, while providing working mothers peace of mind that allows them to find and maintain gainful employment, and ultimately transform the overall trajectory of the family.

Kidogo’s impact to date has however only been measured in terms of improvements in children’s outcomes, the number of mamapreneurs who have been supported, and the number of hours undertaken in providing training and mentorship visits to mamapreneurs (https://www.kidogo.co/impact). There has been no research study measuring the effect of the Kidogo model on women’s economic empowerment at both the caregiver and mamapreneur levels. The proposed research effort, therefore, seeks to evaluate how the Kidogo model can be scaled to enhance the economic outcomes of women living in low-income communities in Kenya. Specifically, the project will: (a) determine the effectiveness of the Kidogo model on women’s (caregivers and mamapreneurs) labor outcomes such as labor force participation, economic productivity, and economic empowerment; (b) identify what works within the Kidogo model, which aspects could be scaled up and what modifications are required; and (c) establish the cost and cost-effectiveness of the Kidogo model to guide policy discussions on the provision of affordable childcare.

### Theoretical framework and theory of change

We conceptualize women’s economic empowerment based on a theoretical framework developed by (Kabeer 1999). Kabeer posits that empowerment is the expansion of people’s ability to make strategic life choices in a context where this ability was previously denied to them. The ability to exercise choice is thought of in terms of three interrelated dimensions: resources, agency, and achievement. Resources include material, human and social resources. Agency refers to people’s capacity to define their life choices and to pursue their own goals. Taken together, resources and agency constitute “capabilities: the potential that people have for living the lives they want, of achieving valued ways of ‘being and doing’ or ‘functioning achievements.’”

**Fig. 1:**
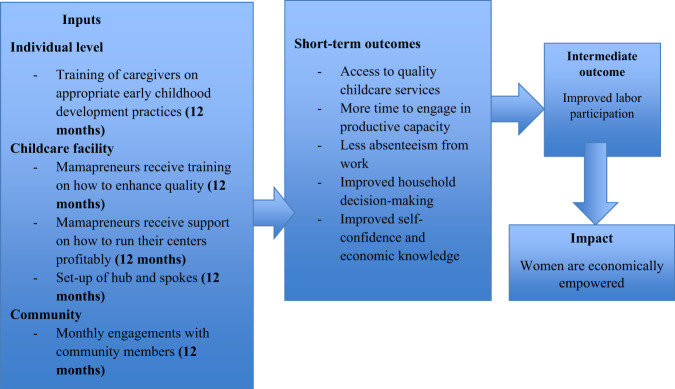
Theory of change illustrating pathways to women's economic empowerment.

The theory of change is based on our experience that the provision of quality childcare services results in women being able to make informed choices about their labor participation. We expect that in the short term, the intervention will improve access to quality childcare services and avail women’s time to enable them to participate in the productive sphere, as they will spend less time in the reproductive sphere (e. g., engaging in child-rearing activities). The childcare centers will provide an opportunity for women to meaningfully engage in economic activities for, at the very least, the required number of working hours in a day. This is essential for them to optimize their income and improve their economic productivity and bargaining power within the household beyond the intervention period. Ultimately, this will contribute to reducing the gender gap in labor participation. In the long term, we anticipate that women will be economically empowered, and the findings will contribute to changes in policy and practice. In creating this change, the program proposes a menu of multifaceted but targeted interventions at three levels: individual, childcare facility, and community (Fig. 1).

The project will adopt a holistic perspective that considers that sustained impacts can only be achieved through significant engagement with caregivers, center care providers, and communities. At the caregiver level, primary caregivers will receive training on how to engage in appropriate ECD practices. At the center care provider level, mamapreneurs will receive training on how to enhance quality within the childcare centers. The training will be delivered to groups of parents and mamapreneurs separately. Mamapreneurs within the community will also receive continued support for running and expanding their micro-enterprises. The broader community will be continually involved as stakeholders through monthly engagements and regular communications about the Kidogo Program activities.

### Gender considerations in childcare

Gender inequality and related discriminatory practices are global problems. Even in nations perceived to have attained basic gender equality, for example, the Nordic countries (Zahidi, 2013) the bone of contention has always been that intangible aspects of discrimination against women have not yet been fully eradicated. For instance, men are more likely than women to be in managerial positions. Moreover, women are more likely to engage in unskilled, low-wage labor, or spend less time in income-generating work and more time in unpaid caregiving work than men. Unpaid care work, which encompasses direct care of persons, housework and unpaid community work includes minding children while also engaging in routine household activities, managing children’s schedules, and taking care of children when they are unwell (Samman et al., 2016). Across the world, women working full-time earn 85% of the wages that men earn for the same work and level of education, thus limiting their ability to access and maintain their families’ needs, including providing children with quality care and education (United Nations Population Fund, 2017). It is against this backdrop that significant efforts are needed to level the playing field insofar as the economic empowerment of women is concerned (Gertler et al., 2012; Payne & Nicholls, 2010).

Apart from the gender discrimination that women experience at the workplace, they are increasingly separated from their children in the first year for a significant amount of time because of employment. Reports show that over half of women with a child less than one year are in employment (Baxter, 2008; Heymann & Kramer, 2009; Payne & Nicholls, 2010) and many may be unable to breastfeed their children for most of the day due to a lack of appropriate space and facilities at the workplace. Low-income laborers are more vulnerable than professional women as the former are less likely to have a private office to express milk or a work environment that is supportive of childcare issues (Amin et al., 2011; Brasileiro et al., 2010; Chen et al., 2013; Ortiz et al., 2004). As such, supporting their childcare needs presents a tremendous opportunity in addressing any issues they may have in childcare provision.

Findings from studies conducted in Mukuru kwa Njenga and Korogocho (Muendo, 2014) show a strong relationship between the provision of childcare services and women’s engagement in work, particularly for low-income employment activities. These studies demonstrated that some of the benefits associated with the provision of childcare services include increased earnings, improved mobility, and an increase in the number of hours worked. In both developing and developed countries, childcare centers have demonstrated up to six-fold increases in breastfeeding and exclusive breastfeeding rates in the first six months, with a stronger impact in developing countries (Imdad et al., 2011). Other benefits associated with the establishment of workplace childcare centers include better employee performance, lower absenteeism rates among employees (Gullekson et al., 2014), and greater peace of mind for mothers. Ultimately, these interventions have shown improvements in women’s participation in the labor force, enhanced improved preparedness for school among their children, and led to increased productivity which consequently results in the country’s economic growth. At the same time, the safety and optimal growth of young children are attained.

## Methods

### Study design

The study reported in this protocol will employ a quasi-experimental design with two study arms, that is, intervention and comparison groups. We will use a mixed-methods approach that combines quantitative, and qualitative data collection methods and records review. We will engage the members of the sub-county and county Health, Education and Social Services Departments to guide decisions on the selection of specific sites for the study implementation. The study sites will be randomly assigned to the intervention and comparison arms with a buffer zone between them to minimize contamination. The participants in the childcare centers within each study site will be purposively selected, and the study arms will be matched based on population size and background characteristics. The intervention arm will comprise women using childcare services provided through the Kidogo hub and spokes while the comparison arm will comprise women using childcare services from other centers that are not supported by Kidogo.

### Study site

The study will be conducted in Nakuru Town West sub-County in Nakuru County. There are five wards in Nakuru Town West sub-County, namely Kaptembwa, London, Rhonda, Shaabab, and Baruti. Kidogo has had substantial coverage and significant influence in Nairobi. For us to evaluate the impact of the Kidogo model effectively, we need to use a “virgin” setting where such an intervention has not been conducted before and therefore, we will utilize the opportunity of Kidogo’s plans to extend their model to other communities including Nakuru. Nakuru is the fourth largest city in Kenya and has been selected because a large proportion of the inhabitants (206,695) live in informal settlements (Kenya National Bureau of Statistics, 2019) with a higher proportion reported in Nakuru Town West sub-County. Similar to Nairobi slums, informal settlements in Nakuru are characterized by high levels of poverty, with the majority of residents engaging in low-paid jobs. Women face challenges with access to quality childcare and have to juggle between family responsibilities (childcare) and engaging in paid work to earn an income to support their families. The community, therefore, stands to benefit from the Kidogo intervention.

### Description of the intervention

Kidogo will establish one to two model centers of excellence for quality childcare with 45–60 children in each. One will be a community hub, while the other will be supported by a private employer. Kidogo will also support 20–40 spokes linked to these hubs, with between 10 and 15 children in each. Spokes will be identified by mapping all childcare centers and recruiting those that will be eligible and willing to participate. Through the social franchising approach, Kidogo will provide training, mentorship, and materials (Starter Kit—a set of materials and resources provided by Kidogo) to the mamapreneurs to help them improve the quality, profitability and expansion of their childcare micro-businesses. Specifically, Kidogo will take mamapreneurs through a 9-month program that offers monthly workshops on an ECD or business-related theme. This will be followed by a bi-weekly, two-hour on-site coaching/mentoring visit by a Franchising Officer.

In recognition of the limitations of in-person interactions occasioned by the COVID-19 pandemic, Kidogo will include a smartphone in its Starter Kit to enable the mamapreneurs to participate virtually in training, receive updates, and have meetings with the franchising team. The internet costs will be borne by Kidogo. The phones will also enable the team to follow up on the business operations of the mamapreneurs in real-time. Primary caregivers in the intervention arm will receive monthly parenting messages.

### Study participants

The primary beneficiaries of this project are mothers with young children below the age of three years. Our sampling frame will include all the women and children who use the childcare services in the hub and spokes, alongside those who use the centers that are not supported by Kidogo. Using the list of childcare centers generated through the mapping activity by the Kidogo team, we will purposively select childcare centers and childcare center caregivers for inclusion in the intervention and control arms. Subsequently we will purposively select primary caregivers receiving childcare services in the sampled childcare centers to be recruited into the intervention and control arms. All the mamapreneurs (intervention arm) and childcare center caregivers (control arm) in the selected childcare centers will be included as secondary beneficiaries.

### Sample size calculation

We anticipate that the intervention will reduce the days of absence among primary caregivers by 50% to 2/24 (8.3%), assuming a rate of absence from work of 17% (4/24) days in a month (unpublished data). A total sample size of 338 (rounded to 340) would be needed to estimate a 50% reduction in the prevalence of absence from work with 80% power, at a 5% significance level, 10% attrition rate, and a design effect of 1.5. We will therefore have approximately 170 primary caregivers in each arm.

The minimum total required sample size for detecting a difference for a two-arm evaluation can be computed as:

$$n = \frac{{\left[ {\left( {Z_{1 - \frac{\alpha }{2}}\sqrt {2\underline {pq} } } \right) + Z_{1 - \beta }\sqrt {\left( {P_1\left( {1 - P_1} \right) + P_2\left( {1 - P_2} \right)} \right)} } \right]^2}}{{\left( {P_1 - P_2} \right)^2}}$$

where

$$\underline p = \frac{{\left( {P_1 + P_2} \right)}}{2}\,{{{\mathrm{and}}}}\,\underline q = 1 - \underline p$$

*P*_1_ is the observed proportion of absence and *P*_2_ is the expected proportion of absence after the intervention.

### Data management

During the process of development of the data collection instruments, we will involve representatives of the different stakeholder groups that operate within the community to reflect the community context better. The stakeholders will be invited to participate in a tool development workshop where they will be asked for recommendations on key questions to include or which ideas to query.

Baseline assessments will be conducted with parents when they join the program. The midline data collection will be completed after 6 months of the intervention implementation. The endline survey will be completed after 12 months. Quantitative tools will be used to establish different outcomes among caregivers. Qualitative tools will be used to establish what works and the scalability of the intervention.

We will use structured interviews to capture information on participants’ sociodemographic characteristics such as age, education, and occupation; outcomes of interest such as labor participation and productivity; and other variables such as autonomy, household decision-making, and economic knowledge. We will measure the impact of the use of childcare centers by establishing changes in women’s labor force participation, the number of hours that they work per week, their income, their household income, time allocated to household tasks, and prospects for career advancement.

To further examine the effectiveness of Kidogo’s Program on women’s labor outcomes, what works within the Kidogo model, and aspects that can be scaled up, we will conduct qualitative interviews with about 50 participants at the different levels, including primary caregivers (mainly women), fathers, and center caregivers from both arms, as well as community leaders and policymakers. Towards the end of the intervention implementation, a small number of key informants will be re-interviewed. All qualitative interviews will be audio-recorded with the participants’ permission.

The costing will be carried out from the implementer’s perspective and will include both incremental start-up costs (such as initial training and awareness-raising activities, investment in infrastructure, and material costs) and implementation costs (such as supervisory and continuous monitoring and mentoring activities, materials, personnel, airtime, transport costs). These costs will be captured from both primary and secondary data. Primary data will be captured through a time-use survey to establish self-reported estimates of the time taken to deliver the intervention activities. Secondary data will be obtained from the implementer’s financial records.

Data will be collected using phone-based and in-person interviews in the case where phone interviews will be rendered not appropriate or feasible. Data will be captured electronically by trained field interviewers (FIs) using tablets and will then be uploaded to the SurveyCTO server. Trained team leaders with oversight from the research team will supervise the data collection exercise. Field team leaders will verify the completeness of the data collected by each FI before uploading it to the server. Discrepancies will be addressed as soon as the field team leader together with the field interviewer involved notes them. To improve the integrity of the data, regular spot-checks and sit-ins will be conducted on about 10% of the sample across each FI during data collection.

Similarly, the quality of qualitative data will be enhanced through the recruitment and training of qualified FIs with experience in qualitative data collection. Pretesting of tools and debriefing will be done to ensure the quality of the data. The interviews will be transcribed verbatim by an experienced transcriber and double-coded (about 10% of the transcripts) to ensure consistency in the application of the codes.

### Data analysis

Thematic analysis will be used to systematically analyze all qualitative data (in line with the key questions) through five stages: familiarization, defining themes, coding, charting, and interpretation. During the familiarization stage, all transcripts will be read in-depth. We will generate codes by interrogating the data and linking narrative content. In the final stages, we will categorize the codes to identify overarching themes. Members of the research team will then meet to jointly deliberate on the themes in order to develop a codebook that they will use to carry out the thematic analysis independently. The transcripts will be coded using NVivo.

A micro-costing approach will be followed and intervention costs will be derived by entering and analyzing data into a customized costing tool created in MS Excel. Cost data will be entered regularly into the tool, accounting for different timings for cost occurrence. Costs will be allocated to the following categories: personnel, materials, infrastructure, capital, supervision, monitoring and mentoring, airtime, transportation, and joint costs. Capital and investment costs will be annualized over their expected useful life. In addition, donated goods or volunteer time will be appropriately accounted for from an economic perspective. Costing data will be summarized and described for each cost category by the target population (mamapreneurs or caregivers). We will use a single summative worksheet to determine the total cost data for each target population. This will be combined with effect data to estimate the incremental cost-effectiveness of the program. Sensitivity analyses will be conducted on the estimates.

Quantitative data will be cleaned and analyzed using STATA version 16. We will use the difference-in-differences (DID) estimator to compare changes in outcomes (such as labor participation) over time between the intervention and comparison groups to estimate impact. The DID gives a stronger impact estimate than a single difference, which only compares the difference in outcomes between treatment and comparison groups following the intervention. Applying the DID method removes the difference in the outcome between treatment and comparison groups at the baseline.

We will use propensity score matching (PSM) where intervention group participants will be matched with the comparison based on their likelihood to participate in the intervention given their observable characteristics. Before applying the DID, we will also test the parallel trends assumption, that is, that the trend in outcomes in the intervention and comparison groups was similar before the intervention. We will first identify the indicators of interest. We will then compare the differences in indicator values from before and after the intervention for the intervention group with the same values for the comparison group. We will use a similar DID approach to estimate changes in primary outcomes such as the proportion of women empowered with regards to their participation in the labor market.

## Discussion

The study presented in this protocol paper aims at measuring the impact of the provision of affordable and quality childcare services on women’s labor outcomes such as labor force participation, economic productivity, and economic empowerment. This impact can only be achieved through sustained change across three dimensions: access to employment and income-generating opportunities which represents resources; women’s economic knowledge and self-confidence which will improve agency; and work productivity which represents achievements. We expect that this work will contribute to building evidence on cost-effective models of childcare provision and impact on women’s labor outcomes in poor urban settings of Nakuru County and which can be scaled up to the rest of the country and the East African region.

Participation in the formal economy is an important vehicle for increasing gender equality and is recognized as the most important route to women’s economic empowerment (Clark et al., 2019). However, in developing countries, and more specifically in sub-Saharan Africa, the proportion of women in formal employment is considerably lower, at 70%, compared to 77% for men (Elson et al., 2000). Moreover, despite a marked increase in women’s participation in the labor market, from 33% in 1990 to 49% in 2018, (International Labour Organization, 2018) disparities exist in the way that labor markets are organized. Women are often concentrated in lower-grade jobs, which affects their financial stability, while men dominate the high productivity sectors. As such, women are unable to commit to social protection contributions due to the largely informal nature of their engagements (Ferrant et al., 2014). Inadequate gender-responsive social protection policies in informal employment further aggravate the gender equality equilibrium (Holmes et al., 2019). This reality compromises the quality, regularity, and formality of female labor engagement, resulting in less control over their time, labor, and mobility (Pedwell, Carolyn; Chant, 2008). The available data indicate an enduring and growing informal economy, which demands innovative approaches to address the productive and reproductive agenda, and ensure that women are engaged in decent work.

### Policy implications

Policy considerations that could result in gender equity include reforms in gender norms and cultural discriminatory practices (United Nations Development Programme, 2014), as well as the recognition of the importance of unpaid care work (Ferrant et al., 2014). Structural changes and deliberate efforts in the legislature, including but not limited to developing, implementing, and monitoring rules, as well as effecting laws and policies that enshrine and promote gender equality (United Nations Development Programme, 2014) could further improve outcomes and the informal economy landscape for women. Such provisions would ensure that women are economically empowered and do not face discrimination in the economic sphere. This includes having equal access to secure, decent jobs and the ability to earn an income and participate equitably in labor markets. It also includes the elimination of economic disparities with the recognition, reduction, and redistribution of unpaid care work. While national-level policy responses are considered vital, practical responses at the grassroot level are key in elevating the position of women in the informal economy.

## Data Availability

The datasets generated during and/or analyzed during the current study will be made available from the corresponding author upon reasonable request.

## Patient and public involvement

No patients will be involved.
